# Calreticulin in Essential Thrombocythemia: StressINg OUT the Megakaryocyte Nucleus

**DOI:** 10.3389/fonc.2017.00103

**Published:** 2017-05-22

**Authors:** Francisco Jose Iborra, Petros Papadopoulos

**Affiliations:** ^1^Department of Molecular Cell Biology, Centro Nacional de Biotecnologia, Madrid, Spain; ^2^Department of Hematology, Hospital Clínico San Carlos, Instituto de Investigación Sanitaria San Carlos (IdISSC), Madrid, Spain

**Keywords:** calreticulin, essential thrombocythemia, myeloproliferative disorders, megakaryopoiesis, thrombopoiesis

## Abstract

Calreticulin (CALR) is a multifaceted protein primarily involved in intracellular protein control processes. The identification of CALR mutations in essential thrombocythemia (ET) and primary myelofibrosis that are mutually exclusive with the JAK2 V617F mutation has stirred an intensive research interest about the molecular functions of CALR and its mutants in myeloproliferative neoplasms (MPNs) and its diagnostic/prognostic value. The recently characterized protein–protein interaction of CALR mutants and MPL receptor has advanced our knowledge on the functional role of CALR mutants in thrombocythemia but it has also uncovered limitations of the current established research models. Human cell lines and mouse models provide useful information but they lack the advantages provided by ex vivo primary cultures of physiologically relevant to the disease cell types [i.e., megakaryocytes (MKs), platelets]. The results from gene expression and chromatin occupancy analysis have focused on the JAK-STAT pathway activated in both JAK2 V617F- and CALR-mutated MPN patient groups, although a more complete analysis is needed to be performed in MKs. Stress related processes seem to be affected in CALR mutant ET-MKs, but the precise mechanism is not known yet. Herein, we describe a culture method for human MKs from peripheral blood progenitors, which could help further toward an unbiased characterization of the role of CALR in ET and MK differentiation.

The role of calreticulin (CALR) has been well documented in the calnexin–CALR cycle in the endoplasmatic reticulum (ER) as a crucial process for proper glycan folding and, beyond that, for calcium homeostasis (1). Additionally, it has been shown that it plays a role in programmed cell death and apoptosis in stress conditions (2) and cancer (3).

Mutations in the *CALR* gene have been identified in myeloproliferative neoplasms (MPNs), namely Type I (del52bp) and Type II (Ins5bp) mutations, the most common ones among essential thrombocythemia (ET) and primary myelofibrosis (PMF) patients in a mutual exclusive pattern with the JAK2 *V617F* mutation (4, 5). However, the molecular mechanism that links *CALR* mutations with the disease is not fully understood. Several studies based on the ectopic expression of CALR WT or each of its mutants in human cell lines (e.g., Ba/F3, UT-7) have resulted in the identification of an important protein–protein interaction with the thrombopoietin receptor (MPL) that seems to be crucial for the cytokine independent growth of Ba/F3 (5–7) or UT-7 (8) CALR overexpressing (O/E) cells. Importantly, this interaction was shown to be fundamental for the thrombocythemia phenotype of transplanted mice with CALR mutant HSC (7). Yet, there are missing molecular events that precede or follow this interaction that should be further characterized. At the same time, it is necessary to define the limitations of the available experimental tools employed for that purpose.

Cell lines are instrumental for the biochemical and signaling pathway analysis of mutants, but in certain cases, they have considerable drawbacks, as the origin of the cell type is critical for the study of physiological or molecular processes and should be carefully chosen. As reported, ectopic expression of CALR mutants in Ba/F3 cells is able to induce cytokine independent growth; however, this cell line does not express MPL (5, 9). This discrepancy was attributed to uncharacterized “stochastic events” that mediated the cytokine independent growth (9) and was taken as a “hint” for the identification of the crucial protein–protein interaction between the MPL and the CALR mutant that activates MPL and consequently induces constitutive JAK2 and STAT5/3/1 activation. Of note, the MARIMO cell line that harbors a CALR mutation (61bp deletion) generating a novel C-terminus domain like all the other reported CALR mutations by +1-bp frameshift is not dependent on JAK2/STAT5 signaling (10), and it does not express MPL (11). These striking differences are useful to explore alternative molecular pathways but are also complicated and somewhat conflicting; they raise critical questions regarding the extrapolation of these results to the human situation and disease, which is by default very complex and heterogeneous. Less-biased approaches such as culture of primary cells [i.e., megakaryocytes (MKs)] have numerous advantages. They are physiologically relevant to the affected cell type (i.e., platelets or MKs in ET or MF patients), and they can be cultured in numbers suitable for downstream applications. Importantly, they allow to study the disease mechanisms per patient, as in many cases, other factors are also critical for the interpretation of the clinical manifestation of the disease, such as gene expression or signaling pathway analysis related to a specific phenotype, genetic predisposition, or gender (12). Importantly, they are not manipulated genetically, avoiding artificial phenotypes (e.g., enhanced or permanent stress responses) that are to be considered when enforced expression is established in immortalized cell lines or primary cells.

## Human Peripheral Blood Progenitors Megakaryocyte-Culture

**Figure 1:**
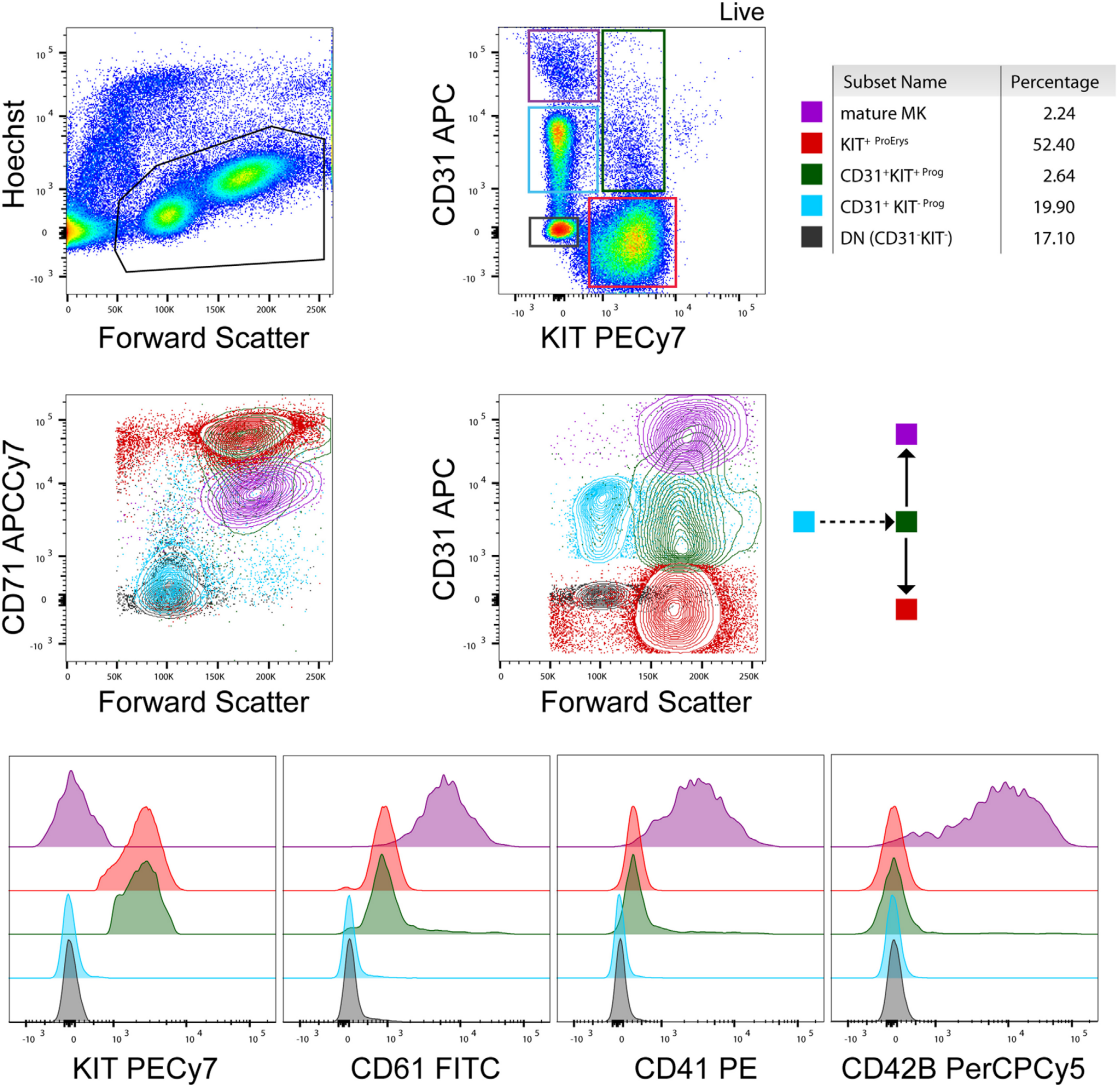
**Flow cytometry analysis of a healthy donor megakaryocyte (MK)-culture at day 10**. A representative flow cytometry analysis based on the expression of KIT, CD71, CD31, CD61, CD41, and CD42b (BD biosciences) surface markers (13). Contour plots and histograms of the mean fluorescence intensity of these markers are included for every population present in the culture [e.g., mature MKs, erythroid progenitors, and early stage progenitors (double negative and CD31^+^ KIT^−^)] as also indicated by the color gates in the contour plots and the cartoon (arrows) indicating cell differentiation. Percentages of cell populations are indicative and depend on the donor and state of health (e.g., % mature MKs is higher in essential thrombocythemia patients than healthy donors) at the time point of collection.

The demand to study the process of megakaryopoiesis *in vitro* in the context of a pathology led us to develop a protocol for the culture of primary MKs from human peripheral blood that can be adjusted to the needs of different experimental approaches (biochemical assays, microscopy, proteomics, etc) (13). Differentiation of the cultured MKs has been characterized based on cell morphology and surface marker expression analysis during the course of the culture (10–14 days that is dependent on the donor). Defined cell populations of erythroid (Erys) and megakaryocytic progenitor cells allow the comparison between healthy and pathologic samples and the identification of lineage-specific discrepancies during the differentiation process (Figure 1). This is extremely important because it permits the study of progenitors and mature MKs simultaneously at different time points during the culture [derived from patients or healthy donors (HD)] that can be subjected to several downstream assays (e.g., sorting, microscopy). Additionally, this type of culture produces platelet particles that can be detected and analyzed by flow cytometry, which resemble platelets from the individual on a fresh sample regarding FSC/SSC and general surface marker expression.

## Subcellular Distribution of CALR and Transcriptional Activity

Expression of CALR is very diverse and given that it is found practically everywhere inside and outside of the cells; differences in expression levels are expected between different cell types and different pathologies. Even more when ectopic expression of mutants is established under different expression levels and kinetics of protein turnover, cell responses can be affected substantially and result in altered proliferation or apoptosis. Consequently, the distribution of CALR can be variable between cell types and upon the same conditions.

**Figure 2:**
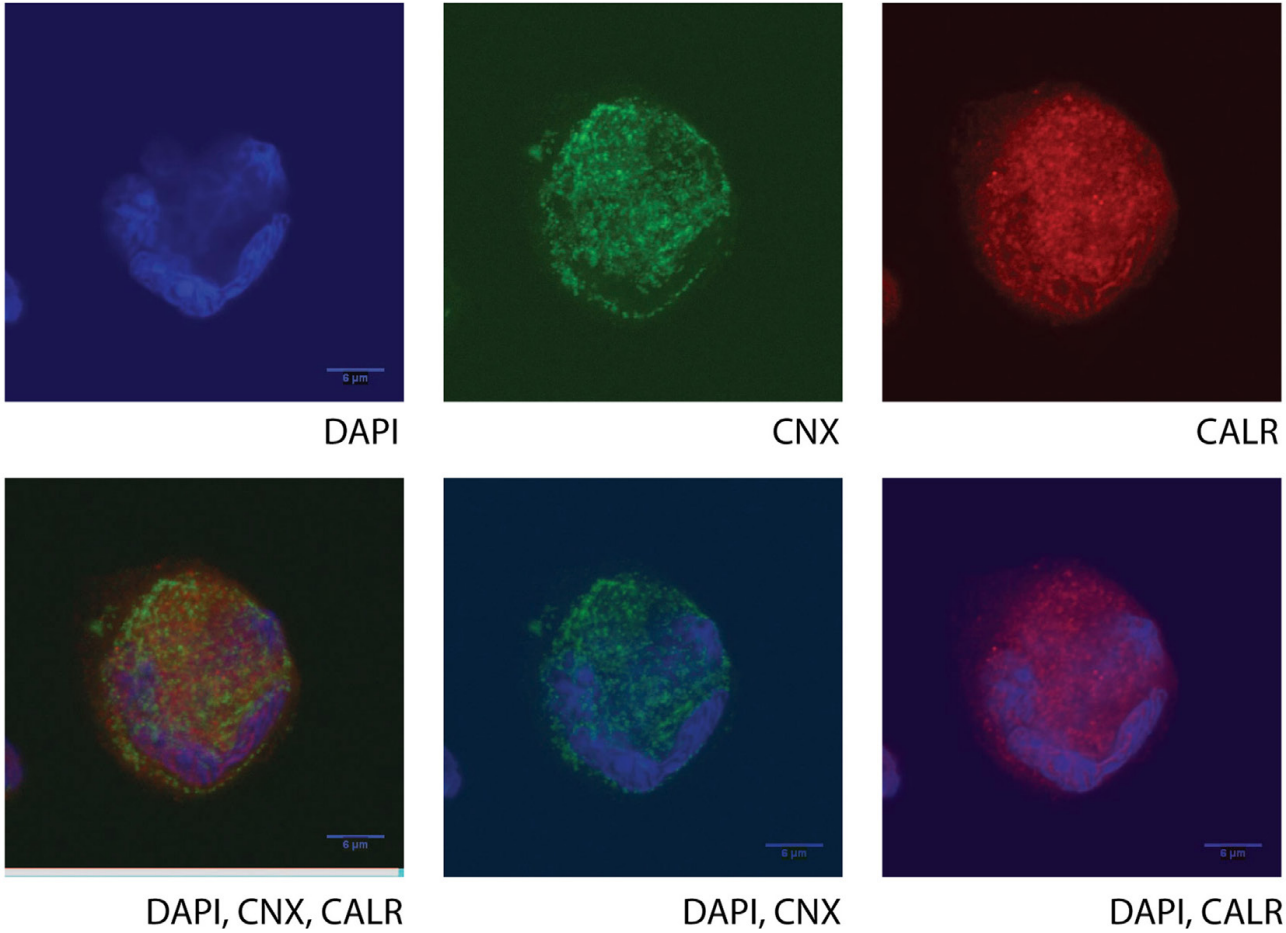
**Confocal microscopy of a human cultured megakaryocyte (MK) from a healthy donor**. Staining for nucleic acid (DAPI, BD), calnexin (CNX, ab31290), and calreticulin (CALR, ab39897, which recognizes an epitope in the N-terminal domain of the human CALR) was performed, and fluorescent signals were detected at the UV channel (DAPI), 488 nm (CNX), or 647 nm (CALR), respectively, on a single mature MK. Different combinations or single detected fluorescent signals are presented in the images. Cultured cells at day 10 (MKs and Erys) were collected and separated by size fractionation on a BSA gradient. The MK fraction was washed in phosphate buffer saline, and the cells were left to attach on a poly-l-lysine-coated glass slide (Sigma). The chosen images are representative of all MKs in the slide, and they show protein staining in one focal plane with a very characteristic morphology (lobular) of the nucleus, typical of the MKs only (polyploid).

As mentioned previously, the commonly accepted expression pattern of CALR is cytoplasmic, membrane and extracellular and thus the efforts for characterization of its molecular functions have focused on these cellular compartments. However, unpublished data from our laboratory suggest that CALR is also present in the nucleus. Confocal microscopy findings of primary MKs from HD (Figure 2) demonstrate the nuclear localization and are in contrast with previous reports in human cell lines [UT-7 (8, 14) or Ba/F3 (6) cells], where CALR is not found in the nuclear compartment. These are important differences that should be taken into consideration at the moment to choose the right experimental model, and they underline the need for further investigation regarding the potential role of CALR in the transcriptional profile of MKs between the different groups of ET (especially between those of JAK2 and CALR) that could affect their differentiation program.

Interestingly, there have been earlier reports about the possible transcriptional role of CALR and its nuclear localization (15, 16). CALR was shown to inhibit androgen receptor and retinoic acid receptor transcriptional activities *in vivo* (15), and specifically, N-CALR reported to interact with the DNA-binding domain of the glucocorticoid receptor (GR), thereby preventing its binding to the glucocorticoid response elements (16). These results implicate CALR in the regulation of gene transcription by nuclear hormone receptors. A very recent study by Falchi et al. (17) has revisited this issue in human erythroid cells (erythroid progenitors) and specifically investigated how the previously described process concerning the shuttle of GR between the cytosol and the nucleus in mammalian cells (18, 19) is mediated by conformational changes of CALR. They used established primary Erys cultures from JAK2 *V617F-*PV (Polycythemia Vera) patients to show that GR is present mainly in the nucleus, and the cells were dexamethasone unresponsive, an effect that was reversed by Ruxolitinib treatment (JAK2 kinase inhibitor) (17). They also implemented confocal microscopy, and interestingly, they did not find significant levels of CALR in the nucleus. So far, a transcriptional role for CALR has not been explored in the context of MPNs (i.e., ET), while the main focus has been the JAK-STAT signaling. The open question is whether the transcriptional profile of MPNs carrying the CALR mutations as compared to the JAK2-mutated MPNs is different and how it is orchestrated in terms of transcription factor and cofactor regulation. A recent report has put forward partially this question and has emphasized the need for further investigation on primary human MKs from patients of *CALR* or *JAK2* ET (20). There is a debate about the flavor of the JAK-STAT signaling in these two groups of patients (20, 21), and gene ontology analysis identifies affected biological processes that are linked to stress conditions in the CALR ET group (20). This is consequent with a cellular response due to the presence of mutated CALR that could trigger several adaptation mechanisms (e.g., transcriptional or translational). However, the possibility that CALR *per se* mediates any type of transcriptional activity is an alternative hypothesis that lacks confirmation at the moment.

## CALR and Stress

Both mutated isoforms of CALR lose their native KDEL signal, and the changes introduced in the negative charge of the C-terminus domain could affect Ca^++^ binding even though only Type I MKs have shown abnormal cytosolic Ca^++^ signals (22). Of note, in Erys cells Ca^++^ flux is induced by EPOR activation (23). The process of Ca^++^ regulated conformational changes of CALR that controls GR shuttling between the nucleus and the cytosol is deregulated in JAK2 *V617F* Erys cells (17) thereby establishing a prolonged stress-responsive gene-expression signature(24) characterized by enhanced proliferation. CALR (C-CALR) in response to Ca^++^ seems to reverse this enhanced proliferation status allowing the initiation of the maturation process by inducing GR nuclear export that has been shown also in murine cells (18). Type I mutation (del52bp) seems to have more pronounced effects as compared to Type II mutation (Ins5bp) since it is observed that it undergoes rapid degradation when over-expressed in cell lines [UT-7 (8), HEL, DAMI, Ba/F3 or 293T (11)] or in transduced HSCs that are subsequently transplanted (7). In the latter case, the phenotype (high platelet counts) was pronounced in the Type I mutation, and the protein levels of the mutant CALR were lower than the protein levels of the Type II (Ins5bp) CALR mutation when over-expressed in HSCs. These results indicate that only low levels of Type I CALR mutant are tolerable by the cell and that this situation is the outcome of a counterbalance mechanism when the mutant protein levels pass over the cell survival thresholds. Although the levels of the CALR mutants are low in cell lines, there is lack of evidence about the actual protein levels and the distribution of the endogenous and mutant protein in primary cells. A study by Kollmann et al. (11) corroborates this observation in human cell lines but it describes higher total CALR protein levels in platelets from MPN patients, whereas in their MK-cultures from CD34^+^ progenitors they did not quantify protein levels. It has been shown previously that CALR has an important role in integrin-mediated adhesion and signaling (25), which are important for platelet activation. Such quantitative changes at the protein levels of CALR in platelets described by Kollmann et al. could have major implications in megakaryopoiesis and consequently in platelet function; however, a clear distinction should be made between different MPN pathologies (PV, ET, and PMF) and their mutational genotype.

Antibodies specific for the mutants show CALR staining basically in MKs of the bone marrow (BM) of MPN patients but they do not provide a quantitative measurement (26, 27). On the contrary to what has been observed in ectopically expressed CALR mutants in cell lines, the majority of ET and PMF BM biopsies had very high intensity signals in MKs and lower in other cell types (27). This could indicate early stage MKs that undergo a process of adjustment of mutant CALR levels under the cell survival threshold, and in order to follow this process, a physiological and unbiased system is needed (i.e., primary cultures).

Endoplasmatic reticulum and mitochondria play an important role in the orchestration of such response mechanisms under stress conditions, although little is known so far about their role specifically in MKs under the presence of CALR mutants and in megakaryopoiesis and thrombopoiesis in general.

## Future Directions

It is interesting to investigate the nuclear levels of CALR in ET-MKs as compared to HD MKs and if quantitative changes at the protein level could be linked to proliferation, apoptosis, proplatelet formation, and platelet function under stress in the different groups of ET. Given that the reported results in cell lines are dependent on certain limitations and often do not correlate completely with each other, future emphasis should be given to the primary cultures (i.e., MKs) from HD and ET patients, which provide an unbiased system, and they are representative of the donor characteristics. Transcriptional profile of human MKs from ET and HD would be very informative and should provide us with insights about the possible transcriptional implication of CALR in the MK differentiation program in steady state and in disease. Identification of markers that could help us understand the biology of ET and ease the management of the patients should be the primary goal of this approach.

## Ethics Statement

Human samples have been processed according to the guidelines of the ethics committee of the Hospital Clinico San Carlos (Madrid, Spain) under the approved project reference (C.P.-C.I. 16/257-E_BS). All research participants provided written, informed consent, in accordance with the Declaration of Helsinki.

## Author Contributions

FI acquired and analyzed the images of confocal microscopy and wrote the article. PP prepared the MK-cultures and processed the samples for flow cytometry and confocal microscopy, performed image analysis and flow cytometry analysis and wrote the article.

## Conflict of Interest Statement

The authors declare that the research was conducted in the absence of any commercial or financial relationships that could be construed as a potential conflict of interest.
